# Skin Colour Does Not Define Ethnicity: Quantifying Variation and Overlap Across Diverse Populations

**DOI:** 10.1111/srt.70343

**Published:** 2026-03-13

**Authors:** Yan Lu, Kaida Xiao, Changjun Li, Michael Pointer

**Affiliations:** ^1^ Leeds Institute of Textile and Colour School of Design University of Leeds Leeds UK; ^2^ School of Computing University of Science and Technology Liaoning Anshan China

**Keywords:** CIELAB colour space, ethnicity, ISSA, perceptual overlap, skin appearance, skin colour, skin colour variation, skin gamut

## Abstract

**Background:**

Skin colour is a prominent human trait historically used to define ethnicity, yet its validity as a classification tool remains questionable.

**Materials and Methods:**

We quantitatively analyse over 14 000 skin reflectance measurements from eight ethnically diverse groups in the International Skin Spectra Archive (ISSA), using a standard colour space designed to model human visual perception. We assess intragroup variation and intergroup overlap through two complementary approaches: individual‐level perceptual differences and group‐level shared gamut volumes.

**Results:**

Results show that within‐group variability in chromaticity and lightness frequently exceeds between‐group differences. At the individual level, 89.4% (95% CI: 81.5%–91.9%) of samples have perceptually indistinguishable counterparts across ethnicities. At the group level, the median shared gamut overlap is 60.5% (95% CI: 54.5%–63.6%), indicating substantial overlap in skin colour distributions. The two methods correlate strongly (*r* = 0.83, *p* < 0.001), confirming robust intergroup overlap.

**Conclusion:**

Skin colour exhibits high within‐group dispersion and extensive between‐group overlap. These findings challenge the use of skin colour as a reliable indicator of ethnicity and underscore the need for objective, data‐driven classification frameworks. They also highlight the complex, continuous nature of human skin variation, beyond simplistic ethnic categories.

## 1 Introduction

Skin colour is one of the most noticeable traits in human appearance and has been used as a primary characteristic for categorising people into racial or ethnic groups [1, 2]. The associations between skin colour and ethnicity have deep historical roots, shaped by centuries of sociopolitical contexts and scientific classification [3, 4]. However, the validity of skin colour to define ethnicity has rarely been critically quantified, largely due to technological limitations and challenges associated with accurately measuring skin colour across diverse populations. Despite the broad recognition of human skin colour diversity, empirical evidence supporting strict links between skin tones and specific ethnicities remains limited.

The widespread acceptance of race classifications based predominantly on skin colour continues to influence daily life, clinical practice, scientific research and technological applications. In daily life, individuals frequently rely on visible skin colour as a primary marker to categorise the race of others and tend to use monoracial categories even in multiracial populations [5]. In clinical settings, treatment decisions and diagnostic reasoning are often implicitly shaped by racial categorisations [6, 7]. Similarly, research across various disciplines, including dermatology, imaging, biology and sociology, regularly associates ‘Caucasian’ with lighter skin pigmentation and ‘Asian’ with more yellowish tones, perpetuating simplistic stereotypes [8, 9]. In dermatology and cosmetic science in particular, such assumptions have historically informed product development, clinical assessment scales and visual diagnostic criteria, despite growing evidence of substantial skin colour variation within conventionally defined groups [2, 10, 11, 12]. This oversimplification extends beyond skin research, impacting broader fields where skin colour implicitly encodes racial identity [13].

Without rigorous empirical validation, this linkage risks obscuring genuine phenotypic diversity and inadvertently perpetuating biases in scientific inquiry across various domains [14]. Furthermore, the reliance on these racial skin stereotypes may embed biases into more recent technological applications such as facial recognition systems, skin‐type classification tools and consumer‐facing algorithms used in artificial intelligence–generated content, consumer electronics and cosmetic product matching [15, 16]. Such persistent oversimplifications raise an essential question: To what extent can ethnic groups genuinely be distinguished by skin colour alone? Answering this question critically informs efforts toward more inclusive and scientifically accurate representations of human diversity.

Efforts have been made to understand the genetic basis of skin colour and its evolutionary adaptation, yet, influenced by earlier classification biases, the complexity of its genetic architecture has been underestimated [17]. Recent genomic research, however, reveals that human skin colour results from a complex interplay of numerous genetic variants interacting with environmental and cultural factors [18]. Anthropological studies further demonstrate that genetic diversity in skin pigmentation is atypically distributed and unsuitable for strict racial categorisation [19]. The new era of genomic research highlights the much more complex nature of skin colour than previously understood and underscores the necessity of accurately assessing skin colour as both a biological and cultural phenotype across diverse populations [3, 18].

Skin colour measurement has a long history, with early approaches relying on subjective visual assessments such as the Fitzpatrick scale [20] and the Taylor Hyperpigmentation Scale [21]. While widely used in clinical and research settings, these visual classification methods are inherently limited by categorical definitions, observer bias and restricted sensitivity to continuous variation in skin colour. The development of non‐invasive measurement technologies, combined with systematic colorimetry approaches established by the Commission Internationale de l'Eclairage (CIE) [22], has enabled objective and precise evaluation of skin colour. Among various systems, the CIELAB colour space established by CIE, has become the most widely used for representing human skin colour [23]. It defines skin tone in three perceptually uniform dimensions: lightness (L*), redness (a*) and yellowness (b*), corresponding closely to biological features such as melanin concentration, erythema and tanning responses. To systematically study global variations, CIE established Technical Committee TC 1–92 in 2013, providing guidelines for skin colour measurement and initiating the International Skin Spectra Archive (ISSA) [24]. ISSA is a publicly accessible database comprising objectively measured skin colour and spectral data collected under standardised protocols. By adhering to uniform measurement standards and instrument calibration procedures, ISSA enables robust comparisons of skin colour data collected globally and addresses measurement variability across instruments [25, 26].

Utilising objective measurements data from ISSA, the present study challenges the traditional race‐based assumptions about skin colour through rigorous quantitative analysis. We examine intragroup variation and intergroup overlap in skin colour across eight self‐defined ethnic groups using the CIELAB uniform colour space. While differences in lightness (L*) and yellowness (b*) are observed between groups, within‐group variability is greater and shows substantial overlap across ethnicities in both chromatic and luminance dimensions. Applying two complementary analytical approaches, we find extensive intergroup overlap. Specifically, at the individual level, the median overlap percentage of samples below the perceptual colour difference threshold ($\Delta E_{ab}^{*}=2$) is 89.4%, indicating that most individuals can have perceptually indistinguishable skin colours from individuals across different ethnic groups. At the group level, the median shared gamut volume is 60.5%, reflecting considerable intersection despite differences in overall gamut size. The strong correlation between individual‐ and group‐level results (*r* = 0.83, *p* < 0.001) reinforces the conclusion that skin colour is not a reliable definition of ethnicity. By providing robust empirical evidence, this study underscores the complexity of human skin colour and reveals the significant variability within—and overlap between—ethnic groups, challenging simplistic racial classifications based solely on skin tone.

## 2 Methods

### 2.1 ISSA

To evaluate the skin colour overlap across ethnic groups, a comprehensive database with extensive, high‐quality measurements, covering variations related to ethnicity, gender, age and body location, is essential. ISSA, established under the guidelines of the International Commission on Illumination (CIE), comprises 15 256 real skin spectra measurements from 2113 human subjects collected between 2012–2024. This database adheres to a uniform measurement protocol to ensure data consistency across different global sites and covers a wide variability in skin characteristics. A more detailed description of the measurement protocols and the database specifications is available in the data descriptor paper [24, 27]. This study includes data from eight ethnic groups, summarised in Table 1.

**TABLE 1:** Summary of subjects, gender, the total spectra by ethnic group: CA (Caucasian), CN (Chinese), SA (South Asian—Pakistani), AF (African), IQ (Middle Eastern—Iraqi), TH (Southeast Asian—Thai), JP (Japanese), AB (Middle Eastern—Arabian).

Ethnic group	No. of subjects	No. of females	No. of males	No. of spectra
CA	352	274	78	2376
CN	370	177	193	2736
JP	118	67	51	944
SA	157	92	65	1289
AF	145	85	60	929
IQ	149	77	72	1183
TH	708	494	214	4112
AB	107	87	20	963

Ethical approval for ISSA was obtained from the University of Sheffield (Reference Number: 9) and the University of Liverpool (Reference Number: IPHS‐1314‐278) and ratified by the other host institutions. All participants provided written informed consent, which included permission to share anonymised data for research purposes.

### 2.2 CIELAB Uniform Colour Space

To compare skin colour appearance, measurements must be expressed in a device‐independent, perceptually uniform colour space. The CIELAB colour space [22], recommended by the CIE and widely used in skin research [23], offers a three‐dimensional representation aligning closely with human colour perception. CIELAB includes three orthogonal dimensions: L* (lightness), a* (red‐green dimension) and b* (yellow‐blue dimension). For skin colour, these parameters correspond approximately to perceived skin lightness, redness and yellowness, respectively. Chroma (C*)—representing perceived chroma—is calculated as the distance from the origin in the a*b* plane (Equation 1). The perceptual colour difference $\Delta E_{ab}^{*}$ is computed using the Euclidean distance between two colours ($L_{1}^{*}$, $a_{1}^{*}$, $\;b_{1}^{*}$) and ($L_{2}^{*}$, $a_{2}^{*}$, $b_{2}^{*}$) in CIELAB space (Equation 2).

(1)

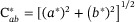



(2)

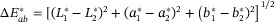




### 2.3 Measures of Intragroup Skin Colour Variation

Intragroup skin colour variation was assessed by calculating the mean and standard deviation for CIELAB attributes within each ethnic group. Additionally, the mean colour difference ($\Delta E_{ab}^{*}$) between each sample and the respective group mean was computed to quantify perceptually meaningful intragroup variation.

### 2.4 Visual Threshold for Skin Colour Differences

Visual thresholds are critical in determining perceptible colour differences, extensively applied in various fields such as dental restoration [28]. Two thresholds are commonly defined: the perceptibility threshold (PT), representing the smallest colour difference detectable visually (just‐noticeable difference, JND) and the acceptability threshold (AT), the smallest colour difference deemed acceptable. Thresholds typically follow a ‘50:50%’ criterion, meaning 50% of observers can detect or accept the difference. Previous studies on maxillofacial skin replications report a PT and AT of approximately 1.1 (SD = 0.5)/3.0 (SD = 0.4) for lighter skin, and 1.6 (SD = 0.5)/4.4 (SD = 1.0) for darker skin, expressed in the unit of $\Delta E_{ab}^{*}\;$[29].

In the current study, the visual threshold serves as a criterion for differentiating individual skin samples. We adopt a suprathreshold based on the PT, set at $\Delta E_{ab}^{*}$ ≈ 2. This threshold ensures that colour differences for different skin types are perceivable by a majority (>50%) of observers, thereby providing a physiologically valid basis for evaluating differences among skin samples and quantifying overlap.

### 2.5 Measures of Intergroup Skin Colour Overlap

#### 2.5.1 By Individual Difference

At the individual level, ethnic group differentiation based on skin colour depends on perceptible differences between samples from different groups. Using the defined PT $(\Delta E_{ab}^{*}$ ≈ 2), we calculated the smallest perceptual colour difference between each sample from one ethnic group and all samples from another group. We then quantified the proportion of these minimal differences falling below the threshold, providing an objective measure of individual‐level skin colour overlap.

#### 2.5.2 By Shared Skin Gamut

At the group level, we assessed skin colour overlap based on the three‐dimensional distribution of skin samples in CIELAB colour space. Because skin colour distribution is irregular, conventional convex‐hull methods [26, 30, 31] may inaccurately represent the true gamut due to potential concavities. Instead, we segmented each group's gamut into discrete 3 × 3 × 3 unit‐cubes. This cube size ensured that the average Euclidean distance between adjacent cubes exceeded the perceptual threshold, creating meaningful colour clusters.

We then refined the segmentation by applying two criteria:

- Removing isolated cubes (surrounded by six empty adjacent cubes).
- Filling gaps (empty cubes completely surrounded by six filled adjacent cubes).

The overlap percentage was calculated by determining the proportion of shared cubes between pair of ethnic groups relative to the total cubes of the reference group's gamut. This approach provided an objective measure of shared skin colour gamut volumes between groups.

#### 2.5.3 Robust Overlap Estimates (Median and Confidence Interval)

Due to skewed distributions, we summarised overlap results using medians as robust measures of central tendency. Sampling variability was addressed by employing bootstrap resampling with 1000 iterations to estimate the 95% confidence interval of the median.

## 3 Results

### 3.1 Description of Data

This study analyses 14 532 human skin reflectance measurements sourced from the ISSA [24, 27]. Participants include individuals aged 18 to 75 years across eight self‐identified ethnic groups: Caucasian (CA), Chinese (CN), Japanese (JP), South Asian–Pakistani (SA), Middle Eastern–Iraqi (IQ), African (AF), Southeast Asian–Thai (TH) and Middle Eastern–Arabian (AB). Skin colour measurements were collected from twelve body positions: back of hand, cheek, cheekbone, chin, ear lobe, forehead, inner arm, neck, nose tip, palm, ring finger and both inner and outer forearm. Due to incomplete coverage across global sampling sites, measurements from the earlobe and ring finger were excluded from further analyses, leaving measurements from ten positions. Table 1 summarises the number of subjects, gender distribution and the total spectra for each ethnic group.

**FIGURE 1:**
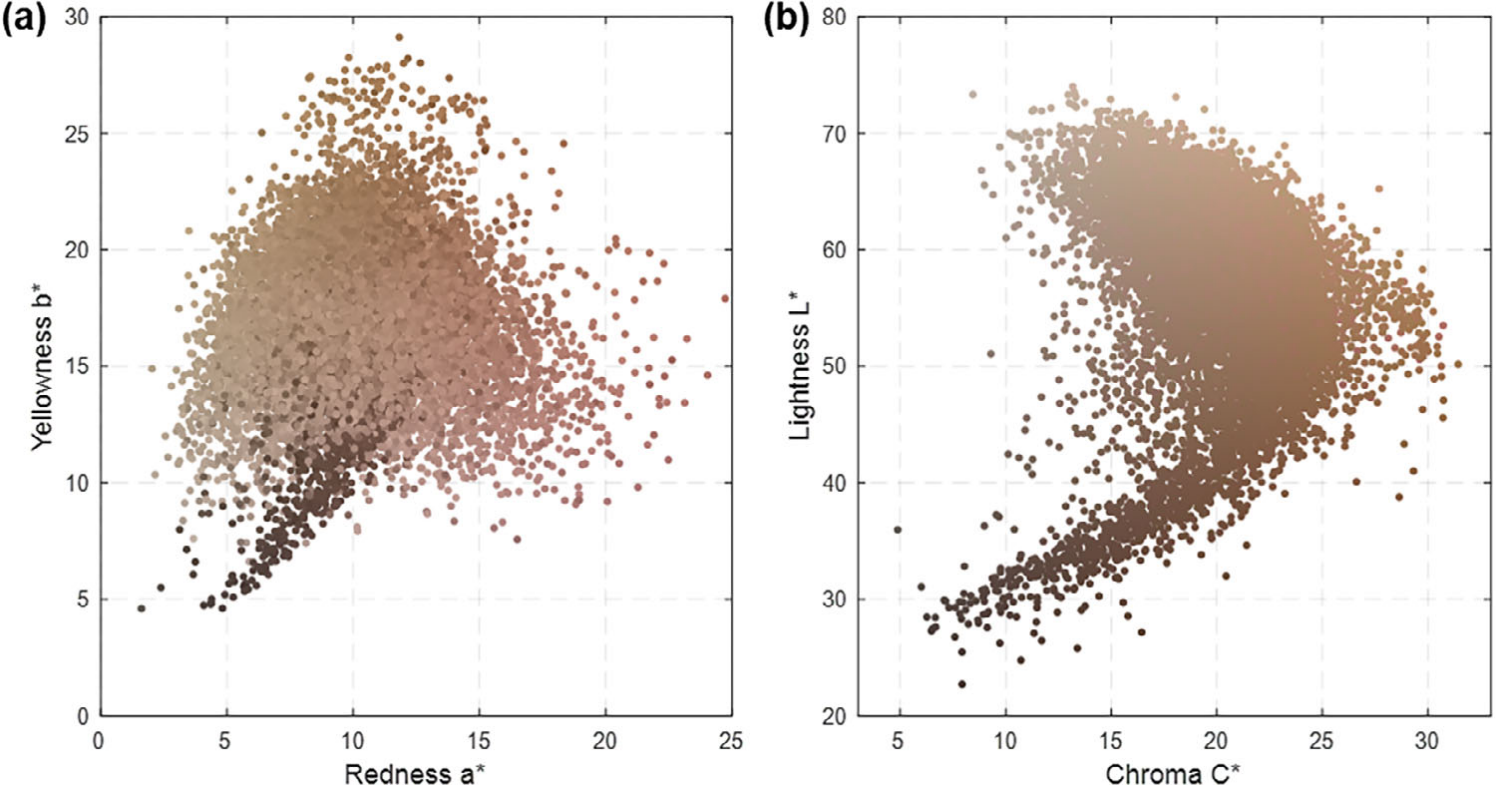
Skin colour distribution (*N* = 14,532) in CIELAB colour space: (a) chromatic a*b* plane and (b) chroma‐lightness L*C* plane.

To facilitate a consistent comparison of colour appearance across diverse samples, skin colour was measured using CIE XYZ coordinates and then converted into the CIELAB colour space, which models human colour perception. The CIELAB parameters, L*, a* and b*, correspond well to the perceptual dimensions of lightness, redness and yellowness, respectively. Figure 1 visualises the skin colour distribution of 14 532 spectra in the CIELAB space: the chromatic a*b* plane (a) and the chroma‐lightness L*C* plane (b). Data point colours approximately represent the actual colour appearance of the corresponding skin samples.

### 3.2 Intra‐Ethnicity Skin Colour Variations

**FIGURE 2:**
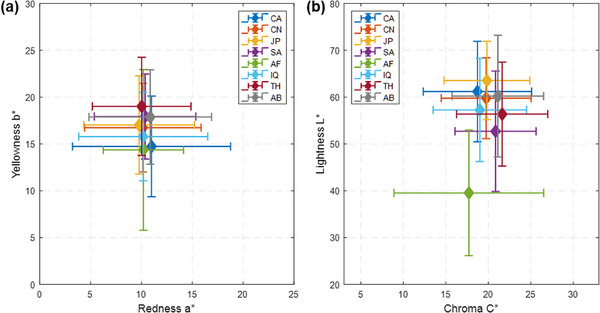
Skin colour distribution of eight ethnic groups in CIELAB space: (a) chromatic a*b* plane and (b) chroma‐lightness L*C* plane. Mean chromaticity values are represented by solid diamonds, with error bars denoting 2 standard deviations. Abbreviations: CA (Caucasian), CN (Chinese), JP (Japanese), SA (South Asian—Pakistani), AF (African), IQ (Middle Eastern—Iraqi), TH (Southeast Asian—Thai), AB (Middle Eastern—Arabian).

Intragroup skin colour variation is assessed using the mean and standard deviation of CIELAB attributes (Table 2). The CIELAB coordinates also allow for the computation of standardised colour difference metrics ($\Delta E_{ab}^{*}$), which are derived as the Euclidean distance in the three‐dimensional space defined by L*, a* and b*. The final column of Table 2 reports the mean $\Delta E_{ab}^{*}$ values and their standard deviation, calculated as the colour difference between each sample and its group mean. This estimate provides a perceptually meaningful measure of intra‐ethnicity variation. Figure 2 shows the dispersed distribution of each group, with error bars representing two standard deviations (approximately a 95% confidence interval).

**TABLE 2:** Mean (± standard deviation) of the CIELAB attributes (L*, a*, b*, C*) and the mean colour differences (± standard deviation) for eight ethnic groups.

Ethnic group	Lightness L*	Redness a*	Yellowness b*	Chroma C*	Colour difference $\Delta E_{ab}^{*}$
CA	61.2 (5.4)	11.0 (3.9)	14.7 (2.7)	18.7 (3.2)	6.3 (3.5)
CN	59.8 (4.3)	10.1 (2.9)	16.7 (2.4)	19.7 (2.6)	5.2 (2.4)
JP	63.5 (4.2)	9.8 (2.7)	17.0 (2.6)	19.8 (2.5)	5.1 (2.3)
SA	52.7 (6.4)	10.4 (2.5)	17.9 (2.3)	20.8 (2.4)	6.4 (3.4)
AF	39.6 (6.7)	10.2 (2.0)	14.4 (4.3)	17.7 (4.4)	6.9 (4.5)
IQ	57.3 (5.5)	10.2 (3.2)	15.8 (2.4)	19.0 (2.7)	6.0 (3.2)
TH	56.4 (5.6)	10.0 (2.4)	19.0 (2.6)	21.6 (2.7)	5.8 (3.1)
AB	60.2 (6.5)	10.9 (3.0)	17.9 (2.5)	21.1 (2.7)	6.5 (3.9)

In the a*b* chromaticity plane, despite the scattered distribution shown in Figure 1a, the mean a* values are tightly clustered across all groups, ranging from 9.8 to 11.0, indicating relative constancy in the redness (a*) dimension. In contrast, mean b* values exhibit greater variability, ranging from 14.7 to 19.0. Although group means are close, the error bars reveal substantial intragroup variation. A considerable overlap in skin chromaticity, in both redness (a*) and yellowness (b*) dimensions, across all eight ethnicities is evident.

In the L*C* lightness‐chroma plane, the distribution of all human skin data typically forms a ‘banana‐shaped’ curve characteristic (Figure 1b). The mean chroma (C*) values vary modestly between groups, ranging from 17.7 to 21.6, primarily reflecting variations along the yellowness (b*) dimension. However, the lightness (L*) level shows a broader range, with values varying from 39.6 to 63.5 among the groups. Although the African group's mean lightness (L*) is notably lower than other groups, its upper range (two standard deviations) overlaps with the range of other populations. Its chroma range also broadly encompasses the ranges observed in all other groups.

Two‐way analyses of variance (ANOVA) was conducted to evaluate the effects of ethnicity as well as gender on skin lightness (L*), redness (a*) and yellowness (b*). The results revealed significant main effects of ethnicity (*p* <0.001) and gender (*p* <0.001) for all three appearance attributes (Appendix Table A1). Across all colour parameters, statistically significant effects were observed given the large sample size. However, effect size estimates indicated that interaction effects were consistently small, and for a*, both ethnicity and gender accounted for only a minimal proportion of the total variance.

Together, these results reveal the substantial individual‐level diversity in skin colour, and the intragroup variability in skin colour is often more pronounced than intergroup differences. In the next two sections, two approaches are applied to quantify these overlaps at both the individual level and group level.

### 3.3 Quantification of Inter‐Ethnicity Overlap by Individual Difference

To assess the extent of inter‐ethnicity overlap at an individual level, we examined whether skin samples from different ethnic groups can be perceptually indistinguishable based on their colour. For each skin sample within an ethnic group, we identified the most similar sample from another group using the minimal $\Delta E_{ab}^{*}$ values. We then quantified the degree of overlap by calculating the proportion of these minimal differences that fall below the just noticeable difference (JND) threshold, set at $\Delta E_{ab}^{*}$ = 2 in the CIELAB colour space.

**FIGURE 3:**
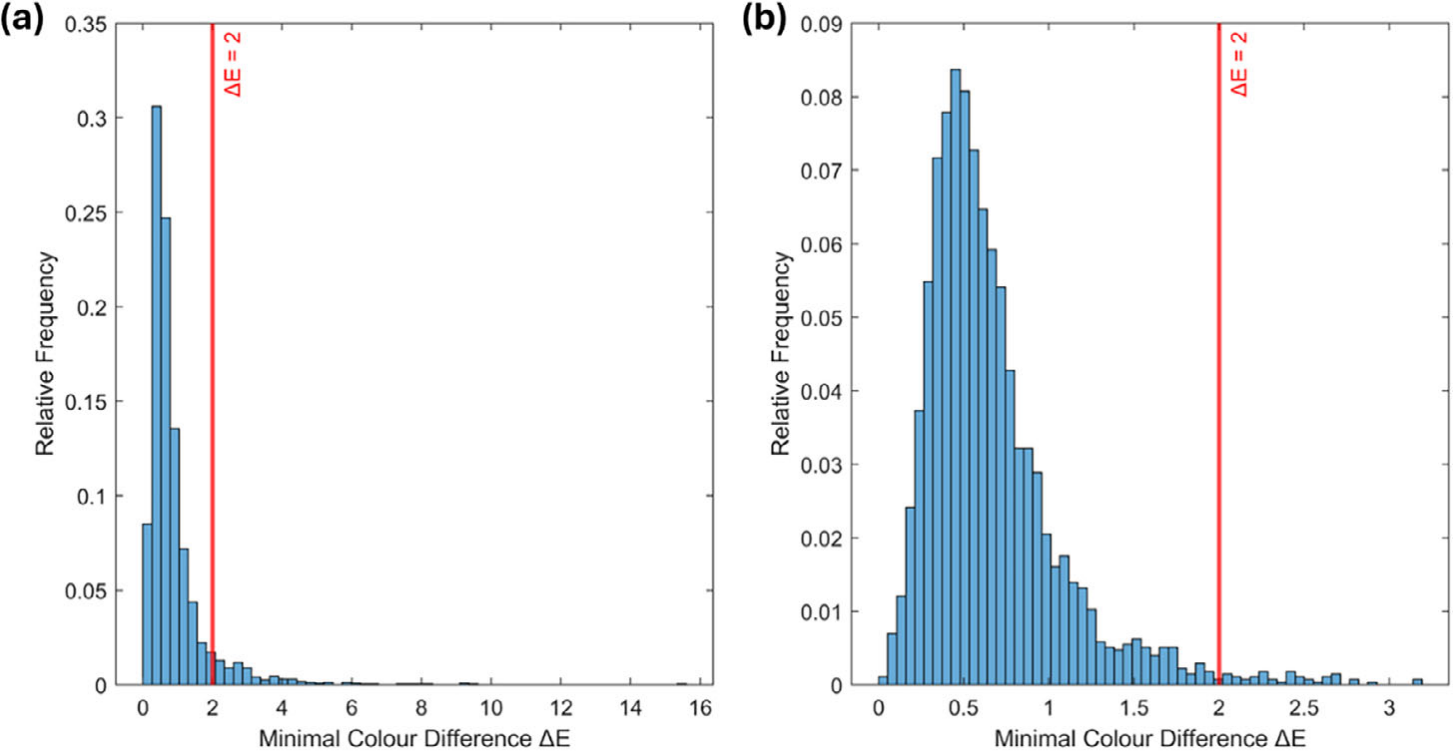
Distribution of minimal $\Delta E_{ab}^{*}$ values: (a) CA samples compared to CN samples, and (b) CN samples compared to CA samples. The red line marks the perceptual threshold ($\Delta E_{ab}^{*}$ = 2).

For example, to determine the overlap between the Caucasian (CA) and Chinese (CN) groups, we calculated the smallest perceptual colour difference for each CA sample relative to all CN samples, and vice versa. Figure 3 illustrates the distribution of these minimal colour differences: the left panel represents CA samples compared to CN samples, while the right panel shows CN samples compared to CA samples. The results indicate 92.3% of the minimal differences for CA samples and 98.4% for CN samples fall below the PT. Thus, virtually all samples from these two groups have perceptually indistinguishable counterparts in the other group, indicating a substantial overlap.

**FIGURE 4:**
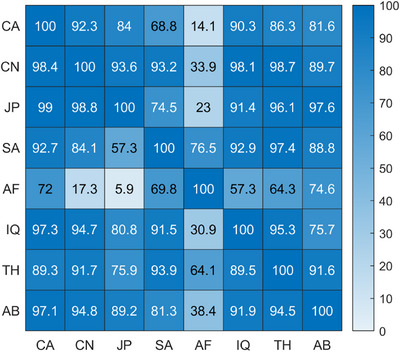
Heatmap illustrating the percentage of perceptual overlap between ethnic groups. Each cell represents the proportion of individual skin samples from the reference group (rows) whose minimal colour differences compared to samples from the comparison group (columns) fall below the perceptual threshold ($\Delta E_{ab}^{*}=2$). Colour intensity ranges from white (minimal overlap) to blue (high overlap). Abbreviations: CA (Caucasian), CN (Chinese), JP (Japanese), SA (South Asian—Pakistani), AF (African), IQ (Middle Eastern—Iraqi), TH (Southeast Asian—Thai), AB (Middle Eastern—Arabian).

To generalise this analysis across all ethnic groups, we constructed a heatmap (Figure 4) illustrating the degree of perceptual overlap between every pair of groups. Rows correspond to reference groups, while columns represent comparison groups. Each cell in the heatmap indicates the percentage of samples from the reference group (row) having colour differences below the PT ($\Delta E_{ab}^{*}=2$) compared to samples in the other group (column). A higher percentage signifies greater perceptual overlap, visualised by a gradient from white (low overlap) to blue (high overlap).

Given the skewed distribution of overlap percentages, we used the median as a robust central measure. To quantify sampling variability and robustness, we applied bootstrap resampling with 1000 iterations to estimate the 95% confidence interval of the median. Across all ethnic comparisons, the median proportion of perceptually indistinguishable samples was 89.4%, with a 95% confidence interval ranging from 81.5% to 91.9%. These findings highlight extensive perceptual overlap between individuals of different ethnic groups.

### 3.4 Quantification of Inter‐Ethnicity Overlap by Shared Gamut

**FIGURE 5:**
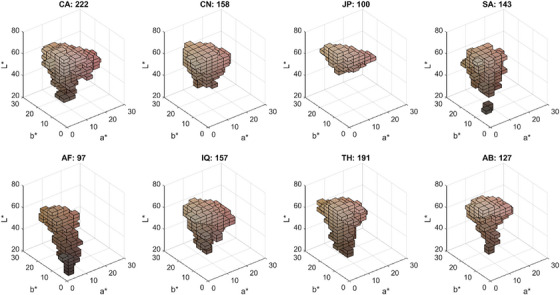
Visualisation of skin colour gamut segmented into 3 × 3 × 3 unit cubes within CIELAB colour space. Numbers indicate total gamut volume per ethnic group. Abbreviations: CA (Caucasian), CN (Chinese), JP (Japanese), SA (South Asian—Pakistani), AF (African), IQ (Middle Eastern—Iraqi), TH (Southeast Asian—Thai), AB (Middle Eastern—Arabian).

Colour gamut represents the range of colours encompassed within a given colour space. To quantify intergroup overlap at the group level, we compared the skin colour gamut of each ethnic group within the three‐dimensional CIELAB colour space and measured their shared volume. Due to the irregular distribution of skin colours (Figure 1), precisely defining gamut volume is challenging. To address this, we segmented the gamut of each group into discrete 3 × 3 × 3 unit‐cubes, ensuring that the average Euclidean distance between adjacent cubes exceeds the perceptual threshold. Figure 5 visualises each ethnic group's skin colour gamut segmented into these cubes, with the number above each subplot indicating the total volume (number of cubes). Notably, the Caucasian group has the largest gamut (222 cubes), while the African group has the smallest (97 cubes).

**FIGURE 6:**
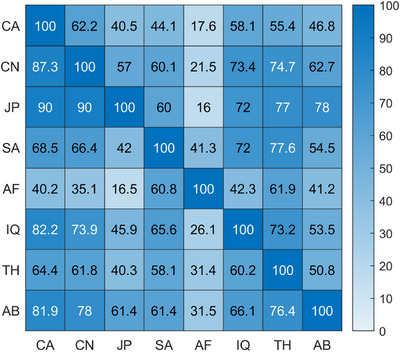
Heatmap depicting overlap percentages between ethnic group gamut, with colours ranging from white (low overlap) to blue (high overlap). Abbreviations: CA (Caucasian), CN (Chinese), JP (Japanese), SA (South Asian—Pakistani), AF (African), IQ (Middle Eastern—Iraqi), TH (Southeast Asian—Thai), AB (Middle Eastern—Arabian).

We quantified the overlap by calculating the proportion of cubes shared between each pair of ethnic groups relative to their total gamut volume. Figure 6 presents a heatmap illustrating these percentages, where each cell shows the proportion of the reference group's gamut (rows) overlapping with the comparison group (columns). For example, 87.3% of the Chinese (CN) gamut overlaps with the Caucasian (CA) gamut, while only 62.2% of the CA gamut overlaps with the CN gamut, highlighting the larger gamut of the CA group. Across all ethnic group comparisons, the median overlap was 60.5%, with a bootstrapped 95% confidence interval ranging from 54.5% to 63.6%.

### 3.5 Consistency Between the Two Approaches

To evaluate the consistency between the two methodologies used for quantifying intergroup skin colour overlap, we calculated Pearson's correlation coefficient between the overlap matrices presented in Figures 4 and 6. This analysis yielded a strong positive correlation (*r* = 0.83, *p* < 0.001), demonstrating a high level of agreement between the individual‐level (minimal colour difference) and group‐level (shared gamut) approaches. These results confirm the robustness and reliability of both methods in quantifying skin colour overlap across ethnic groups.

## 4 Discussion

This study critically examines the relationship between skin colour and ethnicity through the quantitative analysis of over 14 000 skin samples from eight ethnically diverse populations. By combining a reliable database of diverse human skin shades and two complementary analytical approaches, we evaluate both intragroup variation and intergroup overlap. Our findings challenge the traditional use of skin colour as a reliable marker for ethnic or racial categorisation, revealing that within‐group variability often exceeds between‐group differences and that substantial overlap exists across ethnic groups.

Historically, the concept of race implied phenotypic similarity within geographically defined populations. Yet, recent genetic research shows that skin colour is shaped by a complex interplay of genetic diversity, environmental adaptation and sociocultural influences [18]. Understanding the complex nature requires accurate measurement data across global populations. Our study presents data from the ISSA, currently the most comprehensive public database of objectively measured human skin colour. Collected under strict uniform protocols, ISSA provides ecologically valid benchmarks for a wide range of research and applications—from genetics and dermatology to cosmetics and AI. Although not exhaustive of all global ethnicities, Figure 1 demonstrates the observed distribution and range of real human skin colours in CIELAB space, offering a valuable empirical reference. Current classification tools, such as the Fitzpatrick scale [20] and the Monk Skin Tone scale [32], primarily reflect differences in skin lightness and are inadequate for capturing the multidimensional variation in human skin colour across all three CIELAB dimensions (lightness L*, redness a* and yellowness b*).

Consistent with anthropological research suggesting that skin pigmentation is an unreliable marker for other biological traits [19], our analysis reveals substantial variation in skin colour appearance within ethnicities, making it an unreliable indicator of ethnicity. Our analysis also shows that differences between ethnic groups are primarily in lightness (L*), rather than chroma (C*), with yellowness (b*) contributing most to chromatic variation. This aligns with previous research on four ethnicities [26] suggesting that melanin predominantly influences skin lightness and yellowness [33], while factors like haemoglobin and diet have lesser impacts on skin colour modulation [34].

To quantify intergroup overlap, we employed two complementary methods that converged on similar conclusions, with a high correlation between them (*r* = 0.83, *p* < 0.001). The first method assessed individual‐level overlap by calculating the minimum perceptual colour difference between samples from different ethnic groups. Nearly 90% of these comparisons fell below the JND threshold ($\Delta E_{ab}^{*}=2$), meaning most individuals had skin colours indistinguishable from someone in another group. The highest overlaps were observed between Japanese and Caucasian individuals (99.0%), while the lowest occurred between African individuals and other groups—particularly those with lighter skin tones—highlighting the broader phenotypic diversity in darker skin types (Figure 4).

The second method evaluated group‐level overlap through a voxel‐based segmentation of the 3D CIELAB colour space. Unlike previous convex hull approaches [26, 30, 31]—which tend to overestimate gamut volume due to their sensitivity to outliers and inability to represent concave regions (e.g. Figure 1b the ‘banana’ shape in the L*C* plane)—our method counts only populated unit cubes, correcting for isolated outliers and unnatural gaps based on the assumption of skin colour continuity. Although the choice of unit size may affect resolution, this approach offers a more accurate estimation of actual skin colour distributions. Our analysis revealed that Caucasian individuals exhibit the largest skin colour gamut, while African and Japanese groups show smaller ranges—each less than half the size of the Caucasian gamut (Figure 5). This disparity also explains asymmetric overlaps: for instance, 90.0% of the Japanese gamut overlaps with the Caucasian gamut, while only 40.5% of the Caucasian gamut is shared with the Japanese group. Despite these differences, the median overlap across all pairwise group comparisons remained substantial at 60.5%, indicating considerable commonality in skin colour traits across populations.

While prior studies have examined skin colour variation by ethnicity, age or body region [26, 35, 36], the extent of overlap between groups has not been systematically quantified. Our findings contribute to a more nuanced understanding of phenotypic diversity and offer broad relevance across a range of fields—including genetics, medicine [37, 38], digital imaging [39, 40, 41, 42], prostheses [43, 44], cosmetics [45] and AI‐based technologies [15, 46]. Our data and analysis offer a vital reference for researchers and practitioners aiming to develop inclusive systems and products that accurately reflect human skin variation.

We advocate for skin classification systems to move away from race‐ or ethnicity‐based labels and instead focus on empirically grounded measures of skin characteristics. Objective data should be prioritised over subjective self‐reporting methods such as consumer questionnaires [4, 6, 47], especially in applications requiring precise colour matching or representation. Furthermore, research involving global populations must account for within‐group diversity. For instance, studies exploring cross‐cultural preferences for skin pigmentations need to ensure that visual stimuli—whether real facial images [48, 49] or colour‐manipulated images [50, 51]—should reflect both inter‐ and intragroup variation to avoid misrepresentation or reinforcement of stereotypes. Recognising the wide overlap in skin colour is also essential for reducing bias in both human perception and algorithmic decision‐making, particularly in technologies such as facial recognition and classification systems.

This study has several limitations. Self‐defined ethnicity was used as a grouping variable despite being a social rather than biological construct, which may mask heterogeneity within groups. The ISSA database provides standardized and objective measurements collected under controlled conditions, which may not fully reflect the variability encountered in clinical or real‐world settings. In addition, the study focuses on healthy skin samples and does not account for pathological features relevant to dermatological diagnosis. This represents an important direction for future research, as accurate skin tone assessment can influence the visual presentation and differential diagnosis of skin lesions, particularly in individuals with darker skin tones [52].

## 5 Conclusion

This study provides robust empirical evidence that skin colour, while visually salient, is a poor indicator of ethnicity. Through objective measurement and rigorous analysis, we demonstrate that intragroup variation is high and intergroup overlap is extensive. These findings call for a re‐evaluation of how skin colour is used in scientific, clinical and technological contexts, promoting more inclusive, accurate and bias‐aware approaches to understanding human diversity.

## Conflicts of Interest

The authors declare no conflicts of interest.

## Data Availability

The data that support the findings of this study are openly available in The International Skin Spectra Archive (ISSA) at https://doi.org/10.6084/m9.figshare.28228571.v4.

## 1

**Table:** **Table A1** Two‐Way ANOVA examining the effects of ethnicity and gender on skin lightness (L*), redness (a*), and yellowness (b*).

	Source	Df	F	*p*	Partial η^2^
**L***					
	Ethnicity	7, 14 509	2333.431	<0.001	0.540
	Gender	1, 14 509	2784.273	<0.001	0.160
	Ethnicity × Gender	7, 14 509	11.305	<0.001	0.005
**a***					
	Ethnicity	7, 14 509	47.258	<0.001	0.020
	Gender	1, 14 509	237.011	<0.001	0.020
	Ethnicity × Gender	7, 14 509	15.490	<0.001	0.007
**b***					
	Ethnicity	7, 14 509	802.389	<0.001	0.280
	Gender	1, 14 509	31.480	<0.001	0.002
	Ethnicity × Gender	7, 14 509	71.469	<0.001	0.030
